# 4D Porosity Evolution in Additively Manufactured 316L Stainless Steel through *In-Situ* Tensile Testing and X-Ray Computed Tomography

**DOI:** 10.1007/s11340-025-01180-3

**Published:** 2025-04-17

**Authors:** D. Hertz-Eichenrode, H. Talebinezhad, A. Shmatok, R.D. Fischer, S. Bremen, W. Reichert, B.C. Prorok

**Affiliations:** 1 Materials Engineering, Auburn University, Auburn, AL USA; 2 Mechanical Engineering and Mechatronics, University of Applied Science Aachen, Goethestraße 1, 52064 Aachen, Germany

**Keywords:** 4D porosity analysis, Microvoid coalescence, Additive manufacturing, X-ray computed tomography, *In-situ* tensile testing

## Abstract

**Background:**

Many aspects of ductile failure through microvoid coalescence remain elusive due to the challenging spatial and temporal scales it operates on. Experimentally resolving all aspects of the process remains a significant goal of researchers. Much of the current understanding has been derived from post-mortem metallography, leaving key aspects of its evolution undocumented.

**Objective:**

This work builds on efforts using X-ray computed tomography (XCT) characterize voids and their evolution under loading.

**Methods:**

It employs *in-situ* XCT tensile testing on 316L Stainless Steel samples that were constructed by laser powder bed fusion that contain tailored, pre-existing voids with a spatial scale relevant to the growth and evolution stages of microvoid coalescence. Pre-existing voids extended the observation window for monitoring void growth and interaction under loading. They also enhanced fiducial correlation of voids during deformation.

**Results:**

Void populations were found to increase under loading as their deformed dimensions rendered them detectable by the XCT algorithm. Neighboring voids underwent interconnection events by a cleavage process when stress concentrations between them exceeded the macroscopic yield stress. Pores that did not undergo interconnection events were found to revert to their initial size and population after unloading. Finally, the porosity structure before failure was correlated to features on the fracture surface with high fidelity.

**Conclusions:**

This unique combination of *in-situ* XCT tensile testing on samples with tailored void structure enabled new visualization and quantification of void evolution under load as well as strong correlation to the observed stress–strain behavior and post-mortem fracture characteristics.

## Introduction

Most metals and alloys that fail from mechanical loading do so by the process of ductile fracture. This failure mode is complex, dissipating large amounts of energy through the interplay between applied load and various structural characteristics spanning the atomic to microstructural scales. The final stage involves the creation of new surface by the nucleation, evolution and coalescence of voids that eventually cumulate to an unsustainable defect and final fracture [1–4]. Understanding the fundamental aspects of this process has far-reaching implications in predicting the performance and reliability of metallic components.

Despite appearing rather simple in concept, alloy failure via microvoid coalescence is a rather complex process that is not yet fully understood. The process depends on several factors including the microstructure, material properties, applied stress, strain rate and temperature. Recent reviews and references therein provide a good overview [5, 6]. A great deal of theoretical and experimental work has been carried out to provide a fundamental description of the process towards enabling robust predictive capabilities. However, much of the current understanding has been inferred from post-mortem metallographic characterization. The field could benefit from the capability to perform controlled *in-situ* experiments that monitor the internal structure of a material and its evolution under loading. This is challenging as microvoid coalescence operates at multiple spatial and temporal scales making it difficult to experimentally resolve all aspects of the process [7–10]. The objective of this work is to further the understanding of defect identification and evolution at the micro-structural level of internal material features using X-ray Computed Tomography (XCT) in combination with *in-situ* tensile testing.

XCT is now regularly used as an effective inspection tool to non-destructively characterize material microstructure, especially for pore and defect detection [11, 12]. It is capable of quantitatively mapping the size, shape, and location of all resolvable voids [13]. This information can be leveraged in robust statistical analyses that provides insight into understanding how they influence material behavior [14]. Work in recent years has leveraged XCT to visualize and investigate microvoid formation and evolution in various alloys [15–22]. These involved scanning the samples in an XCT, then deforming them in a separate instrument, and finally rescanning them with the XCT. Due to the difficulty in correlating the initial and final positions of individual voids only the average rate of void evolution could be quantified. Others devised tracking algorithms for position correlation but faced challenges with uncertainty and varying results [7, 18, 22, 23]. A more practical approach to void tracking would be to perform *in-situ* deformation by combining a tensile unit with an XCT unit. A handful of studies have taken this approach using an XCT or synchrotron radiation source, more so for composite materials [6, 20, 24–31] and less for alloys [32–35]. Investigating microvoid coalescence in alloys is challenged by the rather small spatial scales that the voids form and evolve on. The spatial resolution achievable with XCT units depends on several factors including the type of radiation source, the attenuation coefficient of the material, sample geometry and volume, detector resolution, and the source-object-detector geometry [14, 36–38]. The nucleation and initial growth of the voids in microvoid coalescence occurs at the submicrometer scale, which is beyond the current resolving capabilities of XCT methods.

The literature cited above has reliably identified defects on the μm to tens of μm scale, capturing the final stages of the microvoid coalescence process. They also involve characterizing those voids that naturally arise from a sample’s particular microstructural features. This presents a challenge in tracking void evolution during deformation, as defect generation can occur unpredictably across the material. Furthermore, once voids reach a size detectable by XCT, they will likely be evolving rapidly with the potential of missing critical stages of their development. An approach to address this challenge is to begin with a sample that contains pre-existing voids. Additive manufacturing, specifically laser powder bed fusion (PBF-LB), has been shown to be adept at generating tailored voids [39–41]. However, these works describe fabrication of rather large voids with spatial scales in the hundreds of μm or larger. Smaller scale voids cannot be tailored directly as the laser spot size is typically on the order of 75 μm or larger. Instead, the laser hatch spacing can be adjusted to generate lack of fusion (LOF) voids. These defects are created when neighboring laser tracks are placed too far apart such that their melt pools are not in full contact [42]. The layered, cross-hatched scanning strategy typical of PBF-LB then renders a 3D array of voids with a spatial scale related to the track gap magnitude. This effort will utilize this approach to create customized LOF voids of a spatial scale relevant to the growth and evolution stages of microvoid coalescence. During *in-situ* XCT tensile testing, these existing voids will act as reliable fiducial markers, enabling more accurate correlation of pre- and post-deformed voids. Additionally, they will extend the observation window, allowing for a more detailed analysis of their growth and interaction under mechanical load.

## Materials and Methods

### Specimen Design, Materials and Fabrication

**Fig. 1:**
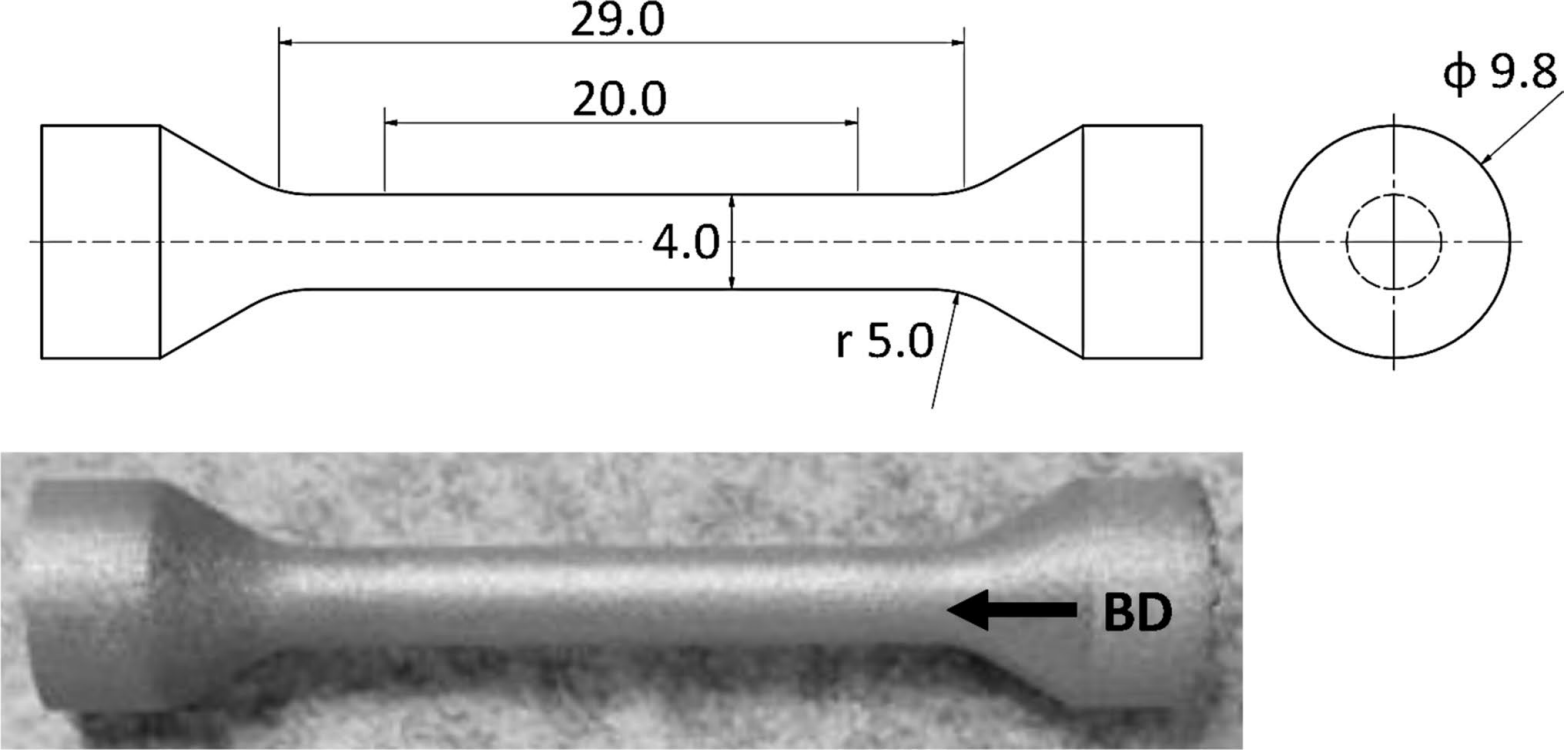
Cylindrical tensile specimen design based on ASTM E8/E8M with dimensions in mm and an as printed specimen before surface preparation. The arrow denotes the build direction

Cylindrical tensile specimens with a diameter of 4 mm were fabricated according to ASTM E8/E8M with dimensions shown in Fig. 1. In order to fit within the confines of the *in-situ* tensile rig, described later, the gauged section was reduced to 29 mm. Specimens were constructed from 316L stainless steel by laser powder bed fusion additive manufacturing (PBF-LB). Fresh 316L powder was acquired from GE Additive with a composition given in Table 1, which adheres to ASTM A276. The particle size distribution provided by the manufacturer indicated that it ranged between 15 μm to 45 μm in diameter. Sample fabrication occurred in a Concept Laser MLab Cusing 100R unit in an inert argon atmosphere. Optimum process parameters were determined by a density optimization study by fabricating 1 cm x 1 cm x 1 cm cubes using a laser power of 90 W, velocity ranging between 400 and 1000 mm/s, hatch spacing ranging between 40 and 120 μm and a layer thickness of 25 μm. Samples were sectioned, mounted and polished using standard procedures before imaging by optical microscopy. The tensile specimens were fabricated with their tensile axis aligned with the build direction.

**Table 1:** Material composition of 316L (1.4404) feedstock powder

Element	C	Si	Mn	P	S	Cr	Mo	Ni
(%)	0.024	0.750	0.920	< 0.045	0.004	17.70	2.31	12.80

Cylindrical tensile samples were manufactured based on two parameter sets: the highest obtained density (HD) and a modest degree lack of fusion (LOF), see Table 2 for parameters. The samples were oriented with their tensile axis parallel to the build direction. Samples were stress annealed at 650 °C for one hour before detachment from the build plate. The sample surfaces were machined to achieve a reduction in diameter of 100 μm to remove the as-printed surface features.

**Table 2:** PBF-LB Parameter sets used in this work

Sample	Power (W)	Velocity (mm/s)	Layer Thickness (μm)	Hatch Spacing (μm)	Energy Density (J/mm^3^)
HD	90	600	25	80	75
LOF	90	800	25	100	45

### Characterization

Density studies were performed by sectioning printed samples along the build direction axis followed by standard mounting and polishing procedures finishing with a 0.25 μm colloidal silica suspension. An Olympus BX51 light microscope and DP73 camera were employed to analyze the cross-sections for density, pore feature, and microstructure assessment. The density was estimated using standard contrast methods [43] and the pore features were categorized by visual analysis. After the density analysis, Adlers Etchant was used to reveal the microstructure.

A JEOL JSM- 7000 F Scanning Electron Microscope operating in secondary electron mode was used to conduct fractographic analysis of the post-mortem samples. Propagation of failure from the initiation point was correlated to the differing topography of the fracture surface and the existing pore structure in the sample. Further analysis was performed through image post-processing including contrast thresholding and correlation to XCT images.

X-ray Computed Tomography (XCT) was employed to nondestructively quantify the internal porosity of the samples. A custom Pinnacle PiXS XCT unit with dual x-ray sources (500kV and 225 kV) acquired images using the following parameters; Voltage 225 kV, Current 109 µA, Power 24.8 W, Binning 2 × 2, Integration time 2250 ms, Number of radiographs 1200, Geometric Magnification 13.3x (SDD/SOD, 1050.0 mm/79.0 mm), with stepped capture strategy. The resulting voxel resolution was 15.04 µm. A 700 μm copper filter was used in front of the source to reduce artefacts such as beam hardening and general noise. All tests and scans were performed at room temperature. VGStudio Max 3.5 was used to reconstruct the XCT scans. This included automated automatic geometrical misalignment and tilt corrections, followed by applying the Iterative artifact reduction algorithm to correct for beam hardening artefacts. The density and attenuation characteristics of 316L were entered and the algorithm was iterated five times using a density threshold of 7.5 g/cm^3^ to distinguish between 316L matrix and voids. Next, a Gaussian adaptive filter was iterated one time to reduce noise using a kernel size of 2 voxels, a standard deviation of 1 voxel, and an edge preservation strength of 0.8. The lower intensity threshold was set just above the background noise and the upper to the peak intensity of the histogram. The porosity analysis was conducted with VG’s EasyPore algorithm. The gray value threshold ranges were chosen as slightly above background for the lower threshold and just below that of the 316L matrix for the upper threshold, the minimum pore volume was set to 3 voxels, and the connectivity set to 26. Finite element simulation of mechanical behavior was also performed directly on the CT scans using the Structural Mechanics Simulation module available with VGStudio. This involved applying static mechanical loading to simulate how the next applied load increment of the tensile tests manifested in terms of the location of stress concentrations relative to the porosity structure.

### Mechanical Testing

**Fig. 2:**
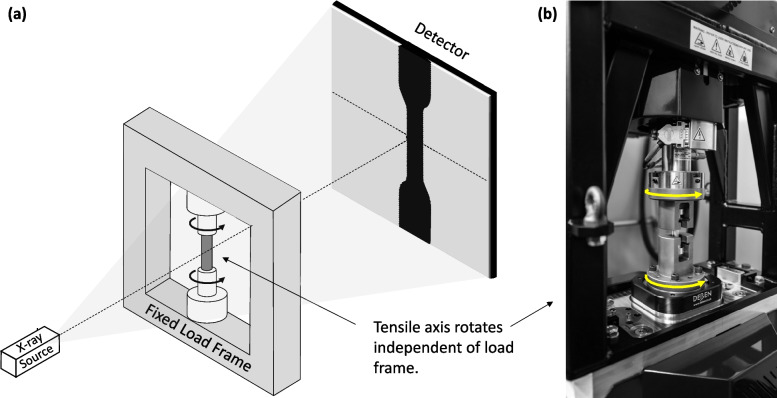
Overall schematic of the experimental *in-situ* XCT tensile testing set-up (**a**) with Deben tensile unit illustrating the load axis movement independent of the load frame (**b**)

Samples were tested until failure on a Deben CT20kN Open Frame tensile test rig with a 100 µm/s crosshead speed following ASTM E8/E8M. Tensile testing was carried out in 2 parts. The first is straightforward tensile loading until failure to ascertain the general tensile behavior of the two PBF-LB parameter sets. These data were used to determine the best approach for the second part of testing involving *in-situ* tensile testing inside an X-ray Computed Tomography (XCT) unit. The Deben load frame was custom-designed for *in-situ* tensile testing within the XCT unit. In this setup, the load frame is fixed and remains stationary throughout the test to prevent interference with the X-ray beam. The tensile axis rotates independently of the load frame, enabling simultaneous CT imaging without obstruction from the dense steel load frame, as illustrated in Fig. 2. The acquisition time for a typical XCT scan is relatively long compared to a standard tensile test. Therefore, to obtain *in-situ* data, the applied load must be held constant during scanning. After each scan, the load is increased to the next level, followed by another scan, and so on. After pre-loading the sample to 100 N, the stress was increased in 1000 N increments until failure. During each XCT scan, the tensile unit was switched to position control to maintain a constant applied strain. An equilibration period was implemented before each scan until significant load changes were no longer observed. XCT images were then acquired, and porosity was analyzed as described previously. To minimize scanning time, the following parameters were adjusted: integration time of 750 ms, geometric magnification of 13.3, and a continuous capture strategy. This resulted in a voxel resolution of 15.04 μm for the *in-situ* scans Fig. 2.

## Results and Discussions

### Initial Characterization

**Fig. 3:**
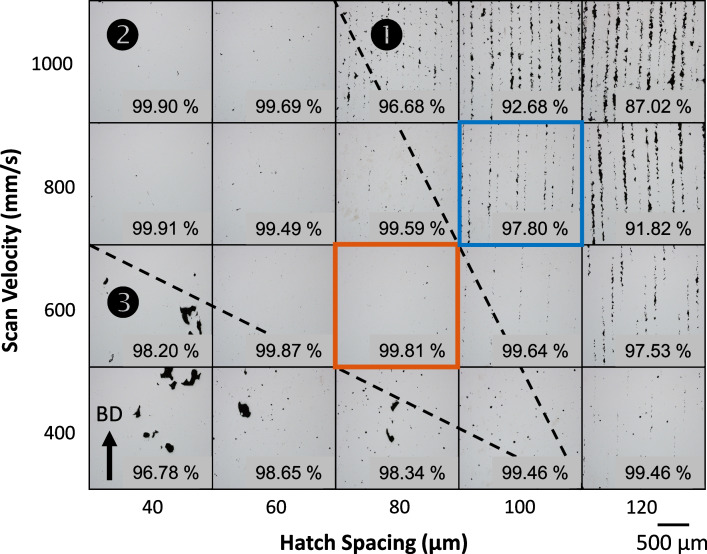
Process parameter optimization matrix showing a typical optical cross-section for each parameter set with labelled density. The red box is considered the optimum density parameter set and the blue box a modest lack of fusion set used in this work

The results of the parameter study to identify optimum density are shown in Fig. 3. Here, the scan velocity and hatch spacing are varied for a constant laser power of 90 W. The parameter space is divided by porosity characteristics into 3 general regions separated by dashed lines. The first is Region 1 that is to the right of the upper dashed line. Here, the LPBF fabrication was performed at relatively low energy density and dominated by conduction mode melting, typically resulting in a lack of fusion (LOF) porosity structure and lower overall density [44]. Region 2 is between the dashed lines and consists of parameter sets that yield the highest overall densities. The melting mode here is defined as transition mode, which is a combination of conduction and keyhole melting processes [45, 46]. Region 3 is below the lower dashed line and contains parameter sets with higher energy density (lower velocity and hatch spacing) that were dominated by keyhole mode melting [47–49]. The porosity structure of Region 3 consists of keyhole pores with the emergence of some metallurgical pores at the higher energy densities. Although there were four high density (HD) parameter sets to choose from, the set consisting of a power 90 W, velocity 600 mm/s and hatch spacing of 80 μm (denoted by the red box and listed in Table 2) was chosen based on surface quality. The blue box highlights the lack of fusion LOF parameter set chosen for this work, also listed in Table 2. This set has a comparable density to the HD set and possesses a well-developed array of LOF pores.

**Fig. 4:**
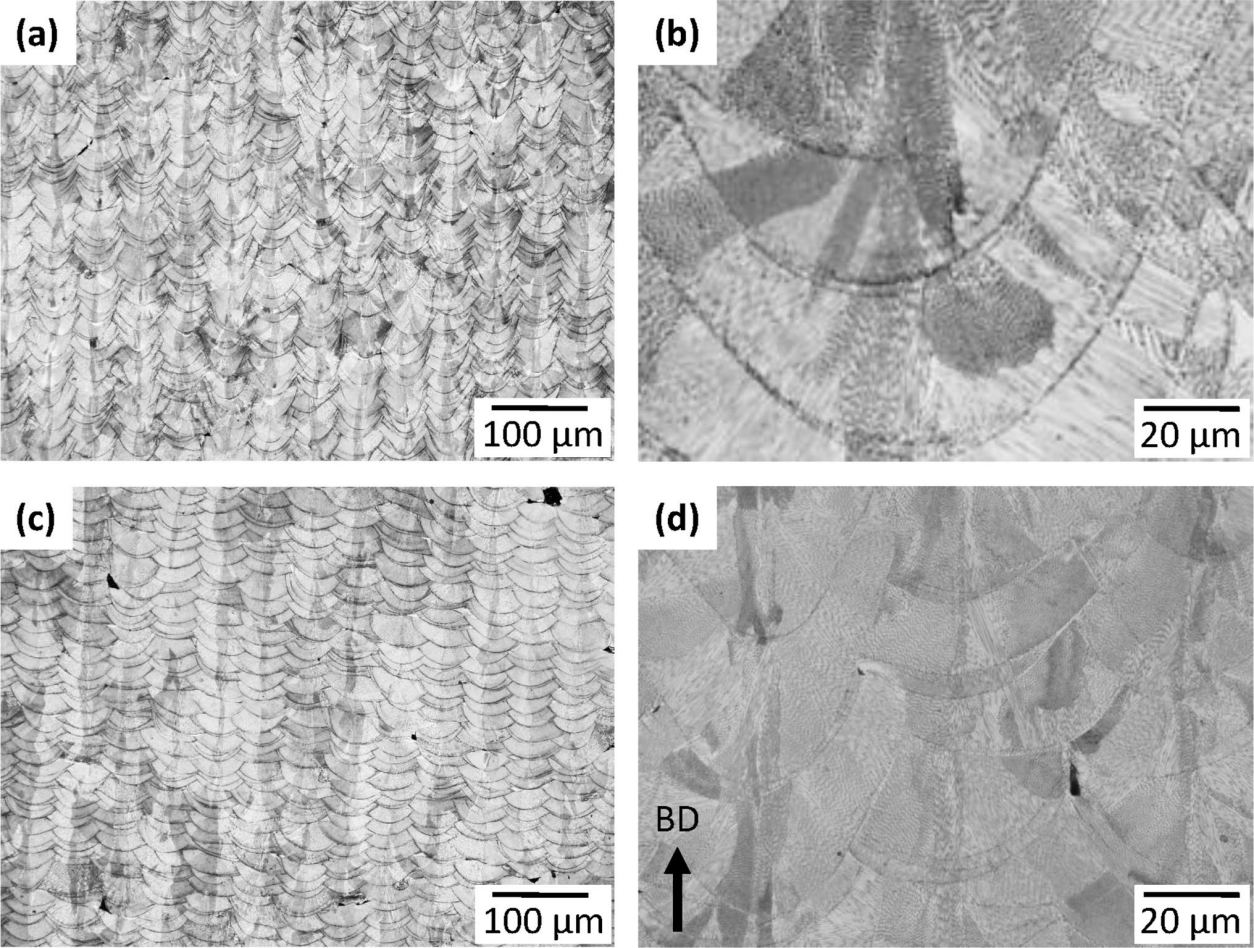
Optical images showing the microstructure at low and high magnification for the HD sample (**a**) and (**b**) and for the LOF sample (**c**) and (**d**)

The typical microstructure of the HD and LOF samples are shown in the optical images given in Fig. 4. This includes both low and high magnification images with parts (a) and (b) showing the HD samples and parts (c) and (d) the LOF samples. Melt pool boundaries are clearly visible and their columnar arrangement in the low magnification images is consistent with hatch spacing of 80 μm for HD and 100 μm for LOF. The higher magnifications also confirm melting modes based on the melt pool depth/width aspect ratio [50, 51]. Part (b) shows a ratio greater than 1.0 indicating transition mode melting in the HD sample while part (d) melt pools are semicircular with a ratio approximately equal to 1.0 indicating conduction mode melting in the LOF sample. The images also show the preferential columnar grain structure that transcends across multiple melt pools resulting from solidification in the presence of a unidirectional heat flux [52].

**Fig. 5:**
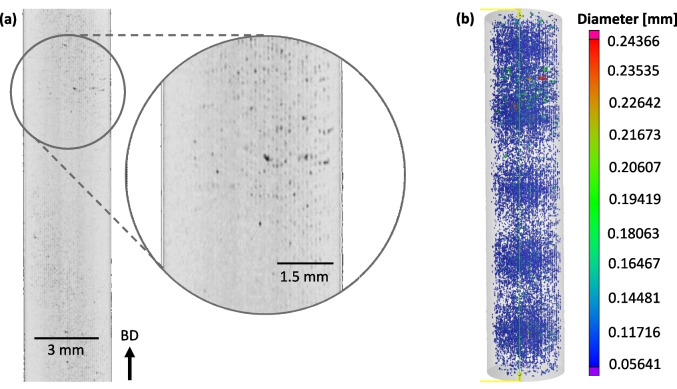
Typical XCT characterization of the LOF samples showing a 2D slice of the 3D reconstructed gauged region (**a**) and the 3D reconstruction with overlaid porosity analysis with pore size denoted by the scale (**b**)

The XCT characterization and porosity analysis for a LOF tensile sample is shown Fig. 5. Part (a) is a 2D slice along the tensile axis of the 3D reconstructed form. The location approximately bisects the diameter along the gauged region length. A zoomed region reveals the array of LOF pores, dark regions. The porosity analysis of the 3D reconstruction is shown in part (b) where some 11,200 pores are identified. The equivalent diameter of each pore is colored relative to the given scale. The pores are relatively uniform in size with the vast majority possessing a radius of approximately 71.4 ± 6.21 μm. In the upper portion of the gauged region, one rather large pore is detected with a radius of approximately 249 μm along with a handful of other large pores of somewhat smaller size. Despite the observed banded or moiré pattern, the pores were relatively evenly distributed. The moiré pattern is an optical illusion caused by the relatively uniform 3D array of LOF pores that mirror the scan strategy. From the observer’s fixed point of view, certain angles have lines of site that are coherent with or align with the array’s periodicity. Here, a single observed pore is aligned with all of the other pores through the volume along that line of site, obscuring them. This gives the appearance of a low pore density. Whereas other lines of site that are not aligned with the array display all the pores, giving the appearance of higher pore density.

**Fig. 6:**
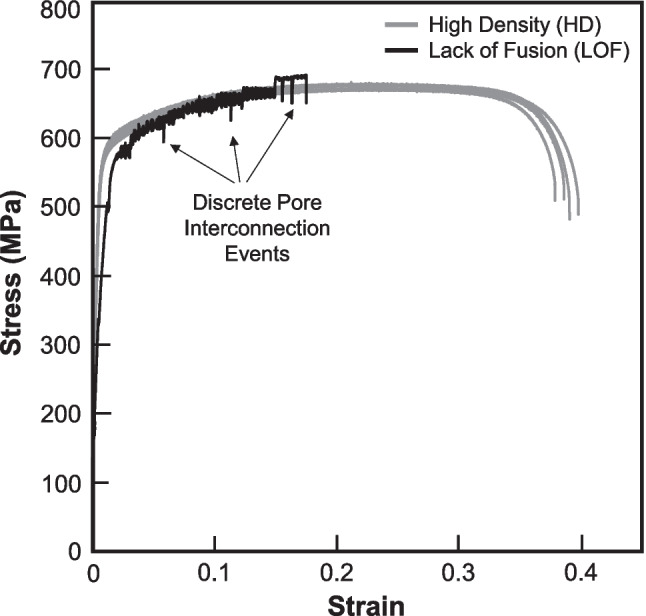
Stress–strain curves of the HD and LOF samples under normal tensile testing

Figure 6 compares the tensile results for the HD and LOF samples. The HD samples displayed near identical behavior and were very similar to the expected behavior for additively manufactured 316L SS [53–57]. The yield strength of the HD samples averaged 544 ± 5 MPa as determined by the 0.2% offset method. Conventionally manufactured 316L stainless steel exhibits a significantly lower yield strength of 220–270 MPa [58, 59]. The tensile strength of the HD samples was 679 ± 5 MPa and lies in the higher range of conventional fabrication, 520–680 MPa [58]. The elongation of 38.87% was somewhat lower in comparison with the lower end of conventional values 40–80% [59]. The differences in mechanical properties with conventionally manufactured samples have been investigated on several occasions and are attributed to microstructural differences such as grain shape and size as well as the higher defect content associated with additive methods [53, 56].

The LOF samples behaved somewhat differently from the HD samples. On average, their measured yield stress was 531 ± 5 MPa. A typical stress–strain curve is given in Fig. 6 for comparison. Only one is displayed as their variability obscures each other’s features as well as the HD samples. In terms of properties, they possess similar yield stress and tensile stress, but significantly lower elongations to failure. Their signatures also show numerous discrete events where load/stress suddenly drops before quickly recovering. These are attributed to pore interconnection events that are characterized in the ensuing *in-situ* testing results.

### *In-Situ* Tensile Testing and Porosity Analysis

**Fig. 7:**
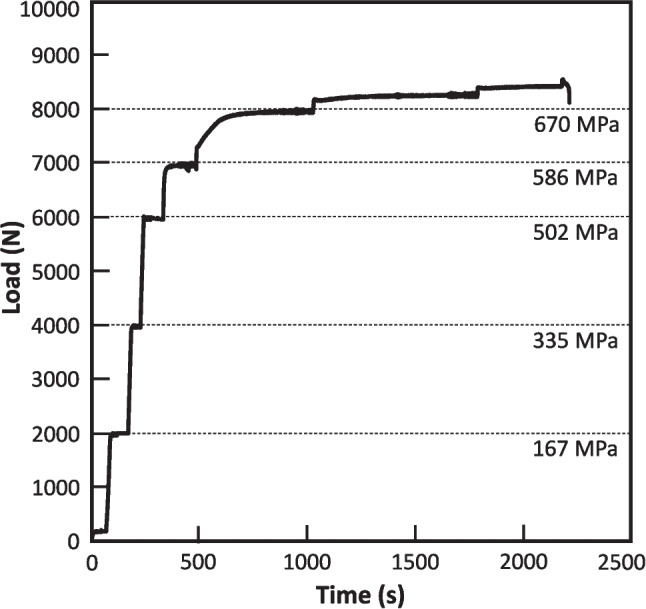
Applied load vs. time signature for an *in-situ* XCT tensile test. The dashed lines represent loads where testing was paused so that XCT images could be acquired

Figure 7 shows the load-time signature of a typical *in-situ* XCT tensile test. The dashed lines correspond to loads where the tensile test was paused to acquire XCT images with associated applied stress values. The equivalent engineering stress is denoted for each load. After a modest preloading of 100 N, the load was increased in 2000 N steps until 6000 N where it was then increased in 1000 N steps. When the load was sufficient to initiate plastic deformation, between 6000 to 7000 N (approximately 500 TO 600 MPa), the pauses required longer times before the load reached an equilibrium condition to initiate the XCT scanning. It should be noted that the unit was operated under displacement control throughout the XCT scan so that the applied strain would remain constant, and the sample features would remain stationary.

**Fig. 8:**
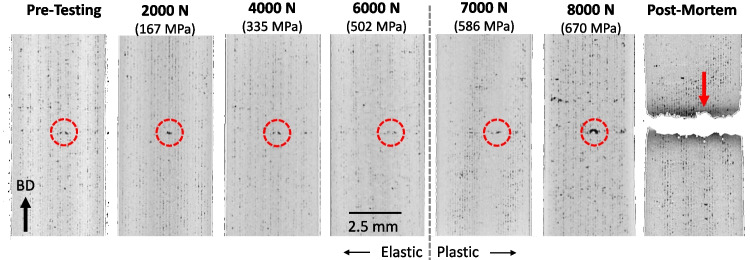
2D slices of a 3D XCT reconstruction of the LOF specimens under various applied load/stress. The dashed circle follows the evolution of the largest pore from scan to scan until failure

The *in-situ* scans were reconstructed into 3D forms for each load level. Figure 8 shows low magnification 2D slices in the gauged region of the LOF sample where the largest observable pore identified in Fig. 5 (b) is located. Seven frames are given in the conditions of Pre-Testing, Post-Mortem and five intermediate applied loads/stress. The largest pore is highlighted by the dashed circle in each slice. It should be noted that it is very challenging to display the exact slice and orientation of a 3D form in seven different reconstructions. In fact, one can observe the vertical moiré-like patterns in how each slice interfaces with the quasi- 3D lattice of LOF pores. Thus, each single cross-section is from a slightly different point of view, causing the pore to appear in somewhat different locations. It is difficult to observe and quantify evolution of the highlighted pore in these images, but a significant increase in size is seen at 8000 N just before failure. The pore is in the direct vicinity of the fracture site and is believed to have caused the circular feature highlighted by the arrow in the Post-Mortem frame.

**Fig. 9:**
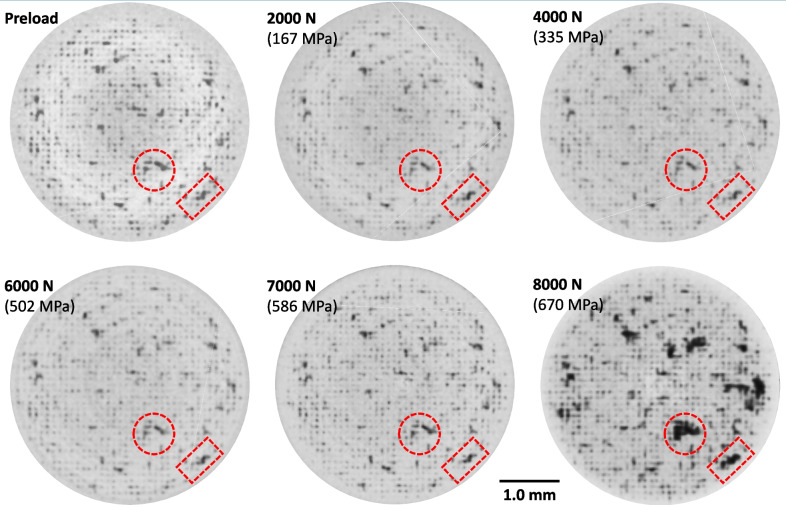
Horizontal cross-sections of the identified failure layer illustrating the porosity evolution as a function of load/stress

Figure 9 shows horizontal cross-sections of the 3D reconstructions at each applied load/stress level of the layer containing the largest identified pore. In all frames, the periodic array of LOF pores is seen by the small, dark grey spots. The larger pores appear more irregular in shape and are presumed to result from sub-optimum processing conditions. The largest pore is highlighted by the dashed red circle. A second pore is also highlighted with a rectangle to serve as a fiducial mark between frames. The porosity structure of the cross-section does not appear to change significantly from the Preload frame to the 6000 N frame. At 7000 N of applied load, a qualitative visual assessment shows that a few of the larger pores appear to modestly increase in size. However, at 8000 N of applied load several of the larger pores significantly increase in size. The geometry of these increases appears to follow the underlying LOF pore array indicating their growth likely occurs via interconnection with neighboring LOF pores.

**Fig. 10:**
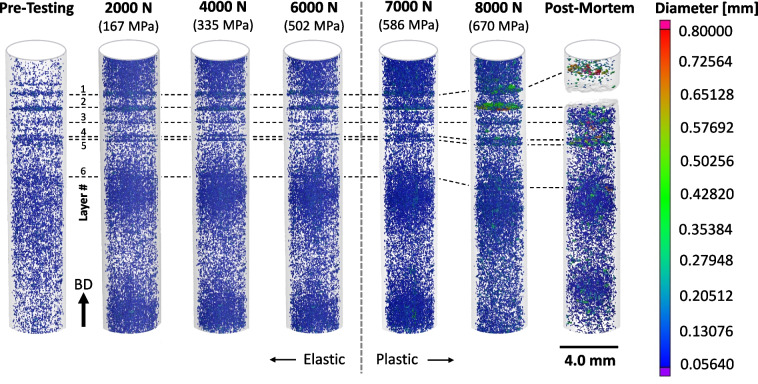
3D visualization of the porosity analysis at each applied load/stress. Note that the size scale is different from that in Fig. 5 (b) to better highlight regions of pore evolution

A quantitative 3D visualization of porosity for the *in-situ* tensile tests is given in Fig. 10. The location of each detected pore in the gauged region, ranging between 11,200 to 16,500 in number depending on the frame, is shown along with their equivalent diameter represented by the given color scale. The moiré fringes seen in Fig. 5 (b) are visible here as well and as discussed earlier are an optical artifact and not a variation of pore density. Several general observations about pore evolution under tensile loading are observed; (1) the number of detectable pores evolves with loading, increasing significantly after 2000 N of load and continues to vary with further loading, see Table 3, (2) the pore population consists of a majority of pores with an equivalent diameter in the 70 to 80 μm range where some significantly evolve once the global yield stress is appreciably surpassed, and (3) there are six discrete layers where pore density appears to be higher, one of which is correlated to failure. These observations are analyzed by considering the population of detected pores from each applied load/stress frame in the following subsections.

**Table 3:** Measured pore characteristics and statistics for the LOF sample

Parameter	Unit	Pre-Testing	2000 N 167 MPa	4000 N 335 MPa	6000 N 502 MPa	7000 N 586 MPa	8000 N 670 MPa	Post-Mortem
Mode	(μm)	73.99	84.01	83.98	84.05	83.96	83.94	74.53
Mode Frequency	(%)	38.57	61.11	59.84	59.90	59.82	55.52	32.33
Detected Pores	(#)	11,203	16,564	16,801	15,541	13,998	11,048	11,010
Change After Load Application	(#)	–	5,361	237	− 1,260	− 1,543	− 2,950	− 38
	(%)	–	48%	1%	− 7%	− 10%	− 21%	− 2%
Accumulated Porosity	–	1.000	2.009	1.957	1.963	1.959	1.900	0.980
Tracked Pore Equiv. Size	(μm)	249	255	256	548	612	658	–

Before discussing the pore evolution observations made in Fig. 10, it is important to consider the types of changes that can occur in the pore population during loading. These include new pores being generated, existing pores changing shape under loading, and two or more pores combining or interconnecting into a single, larger pore. All three are readily possible and have the opportunity to evolve in both number and size during loading. This evolution can be observed by analyzing the information contained in their individual size-frequency distributions or histograms.

**Fig. 11:**
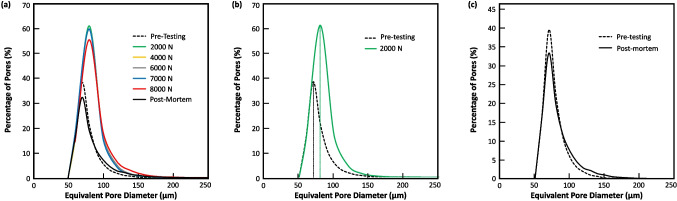
Comparisons of pore size histograms for the various loading segments showing (**a**) all histograms, (**b**) changes after the initial 2000 N load application, and (**c**) the resultant change from Pre-testing to Post-mortem

Figure 11 (a) shows the histograms for the different applied loads/stresses on an LOF sample. Each distribution is positively skewed where the mean is greater than the median, indicating that the population is weighted towards smaller sized pores. This is no surprise for two reasons. First, the LOF samples are generated by employing laser-scanning parameters that create a large, 3D array of similar sized pores based on periodic gaps between melt pool tracks. The second involves the dependence of the pore detection algorithm on the XCT voxel resolution. As mentioned in Sect. 2, the *in-situ* XCT scans had a voxel resolution of 15.04 μm, which limits pore identification to 2 to 3 voxel spacings. Thus, the distributions all begin near the 50 μm equivalent diameter range. Another aspect of the histograms is the dominant pore size population, which is the mode, or more simply put, the pore size that occurs most frequently. Finally, there is the area under the histogram. This area is essentially the summary accounting of porosity determined by the product of the number of pores and their size. An appropriate term for this area is the accumulated porosity (AP). Both the number and size of pores play an independent role in determining its magnitude. As Fig. 11 uses the equivalent pore diameter, it can be considered that the AP is analogous to a measure of porosity volume, since the equivalent diameter of each pore can be easily converted to an equivalent volume. However, this volume is a dependent variable computed from the equivalent pore diameter. The AP can be influenced by changes in the number of pores with only negligible changes in equivalent diameter and thereby equivalent volume. Thus, we will refrain from equating AP with porosity volume.

Under loading, the AP can be influenced by the three mechanisms discussed above. The addition of new pores will alter the histogram by increasing the frequency of certain pore sizes. Newly formed porosity in alloys under loading is typically associated with plastic deformation mechanisms that begin at rather small sizes. Any appreciable addition of new pores will increase the AP through higher frequencies of smaller pores. This would shift the histogram upwards at smaller pore sizes and further skew the distribution in the positive direction. Individual pores that change shape during loading will likely involve elongation in the tensile direction. The number of pores will remain unchanged while the equivalent diameter will increase accordingly. The effect on the size-frequency distribution would be a shift of the mode to a larger size and an increase in AP, both commensurate with the degree of elongation. The mode shift is expected to be the more prominent of the two. When a pore sufficiently increases in size to be promoted, it is removed from its bin and placed in the next largest bin. Whereas its actual increase in size is modest in comparison. The effect on AP can only be connected to the modest size increase as the number of pores remain the same. Thus, the effect on the histogram would be a shift of the mode to the right with only nominal changes in AP.

In the third case of neighboring pores interconnecting, there is a competition between the two independent variables determining the magnitude of AP. Here, one must consider two types of interconnection examples to explore their interplay: (a) two individual, neighboring LOF pores of similar size interconnecting versus (b) a significantly sized pore acquiring a small, neighboring pore. In case (a), the actual effect on the pore population would entail removing two pores from their respective size bins and adding one pore to a larger size bin. On an individual basis, this would have the effect of reducing the mode frequency but not its equivalent size, while increasing the frequency of a larger size bin. For the LOF samples in this work, the effect on AP would be modest as this swaps the product of 2 pores of similar equivalent diameter with the product of 1 pore that a little more than twice the equivalent diameter of the consumed pores. However, the AP would increase with increasing distance between the interconnecting pores. The overall effect of case (a) events occurring in appreciable number would be a reduction in the mode frequency and an increase AP magnitude.

In case (b), a small pore is incorporated into an appreciably larger pore. This would entail removing the smaller pore from its size bin. Given the significant size difference, the larger pore would only acquire a negligible increase in equivalent diameter, where the likelihood of its promotion to the next size bin is small. For the LOF samples this would involve a reduction in mode frequency for the smaller pore’s bin and only a negligible effect on the AP magnitude. For the AP to increase in this case, the large pore would need to interconnect with multiple small pores. Thus, wide scale events of large pores interconnecting with one or just a few smaller pores would cause a reduction in mode frequency while the AP remains relatively constant. Using this framework, the observations made in Fig. 10 and pore size histograms in Fig. 11 can be better analyzed.

*Evolution of the Total Number of Detected Pores with Loading:* Fig. 11 (a) shows histograms of pore size populations at the different applied loads/stresses. Each curve in part (a) begins at an equivalent pore diameter of approximately 50 μm. There is no doubt that a smaller pore population exists; however, as previously mentioned pore detection was limited by XCT voxel resolution. The pre-testing scan (dashed line) indicates that the pore population had a mode, or dominant frequency, of 73.99 μm with the population of smaller pores dropping more sharply than the larger pores. The AP or area under the histogram is assigned the value 1.000 units to more directly compare it with other applied loads. The number of identified pores in the Pre-Tested porosity analysis was 11,203, see Table 3. After the applied load was increased to 2000 N, the number of detected pores increased by 5,361 to 16,564, just over 48%. This matches the qualitatively observed difference between these two frames in Fig. 10. The comparison of their histograms in Fig. 11 (b) also shows a significant increase in pore frequency. This rather large surge in detected pore population is not attributed to newly generated pores, but likely to existing LOF pores that became detectable by the algorithm with their elongated size under loading.

As the load is further increased, the number of detected pores increases to a maximum of 16,801 at the 4000 N scan before decreasing at higher loads, see Table 3. After 6000 N of load, the number of detected pores decreases by 1,260 followed by another 1,543 at 7000 N. This trend continues with a further decrease of 2,950 at the 8000 N load and finally down another 38 pores to 11,010 after failure. The decreases in detected pores can only be interpreted as pore interconnections, where two or more pores become interconnected through inelastic deformation under loading. This interconnection process appears to be under way with the initial decrease of 1,260 pores at 6000 N. At this load, the global applied stress is 502 MPa, which is somewhat below the measured global yield stress of 531 ± 5 MPa. However, stress concentrations at the pore surfaces are likely exceeding the yield stress locally, enabling inelastic deformation to occur. Once the global yield stress is reached and surpassed at 7000 N and 8000 N of applied load, the number of detected pores decreases at a higher rate as pore interconnection events accelerate. After failure, the number of detectable pores reduced to a value below the initial state.

*Evolution of Pore Population with Loading:* The histograms shown in Fig. 11 (a) reveal further details of how the pore population evolved under loading. As previously mentioned, the mode of the pore population in the Pre-Testing histogram was 73.99 μm with a frequency of 38.57%. After the first applied load level of 2000 N (green line) there is an appreciable shift in the dominant pore population to a larger size, see Fig. 11 (b). The new dominant pore diameter increases by approximately 10 μm to 84.01 μm. This includes a 1.6 factor increase in mode frequency to 61.11%. The 2000 N pore histogram returns to the Pre-Testing curve at an equivalent diameter of approximately 150 μm, indicating that this load range does not appreciably affect the population of larger pores. This major pore population shift under loading results in a doubling of the accumulated porosity (AP), or the area under the histogram curve, see Table 3. The 48% increase in the number of detected pores is responsible for the dramatic increase in pore frequency. Although frequency plays a significant role in the doubling of the AP, the shift in dominant pore size can also be attributed to elongation of the pores in the tensile direction.

The next three applied load levels of 4000 N, 6000 N and 7000 N exhibit histograms that are so similar they are difficult to separate. The dominant pore diameter, mode, remains very similar to that of the 2000 N applied load, indicating there was only negligible size evolution in the majority of detected pores. However, their mode frequency does decrease a few percentage points from the 2000 N histogram. This occurs first at the 4000 N load and remains relatively constant at the higher loads. This is confirmed by observing the area under each of the histograms, or AP, which also remains relatively similar between all three applied loads. This decrease, along with the decrease in the number of detected pores, indicates that only a small percentage of pores are undergoing any changes. Those that do are likely involved in interconnection events that result from localized stress concentrations. Being so few in number, they have little effect on the overall porosity population. Like the 2000 N applied load, the population of pores on the order of 150 μm and larger do not appear to be significantly affected in these load ranges.

A more significant change in pore population is seen after application of the 8000 N load. Like the previous three applied loads, the dominant pore diameter remains relatively the same, but their population decreases appreciably. This is coupled with an increase in the population of pores approximately 100 μm and larger. At this load, the global applied stress significantly exceeds the yield stress indicating there is ample driving force for the missing dominant sized pores to become interconnected with a larger pore or accumulate together with other similar sized pores into a single larger pore. The degree to which these events are happening is reflected in a reduction in the AP as compared to the previous three applied loads, see Table 3.

After specimen failure, the Post-Mortem pore population histogram reverts to a population fairly similar to the Pre-Testing histogram. Figure 11 (c) gives a direct comparison between the two. First, the dominant equivalent pore size has returned to the Pre-testing value, but at reduced population. The relaxed load and associated recovery of stored elastic strain has returned many of the pores to their undeformed dimensions, which has also rendered some pores no longer detectable by the porosity algorithm. Second, a portion of the dominant pore size is appreciably reduced. However, this is balanced by an increase in the population of pores approximately 90 μm and greater. This confirms that some of the pore population has been incorporated into larger pores. Additionally, the final accumulated pore population is lower than the Pre-Testing histogram. It is expected that it should be somewhat lower as some of the pore population is consumed in the creation of fracture surface. However, the difference may also depend on the change in both pore number and volume.

*Discrete Defect Layers:* The 3D porosity visualizations shown in Fig. 10 reveal the existence of six discrete defect layers within in the gauged region. From the viewer’s perspective, they are disc-like regions where the density of the ~ 70 μm (blue) pores appears to be appreciably higher than in other regions. This may be an indication that these layers were manufactured under sub-optimal conditions. The dashed lines trace their locations as the tensile sample deforms under load. Note that the pore visualizations are centered on Layer 2 as it appears to be directly related to the fracture site. Thus, the other layers shift away from the fracture site as the sample elongates under tension, especially after significant plastic deformation and fracture. The defects in Layer 2 appear to have evolved significantly in the 8000 N frame with multiple pores growing to a significant size relative to any other area in the gauged region. After failure, the postmortem frame indicates that certain pores also began to grow significantly in the other identified layers containing higher porosity.

**Fig. 12:**
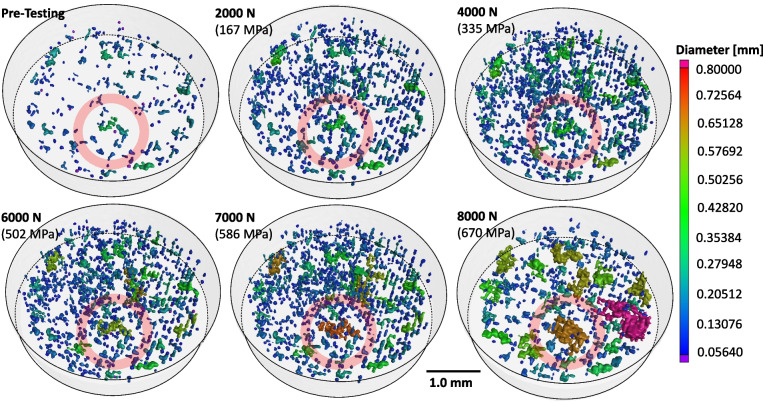
3D reconstructions of defect Layer #2 illustrating porosity evolution as a function of applied load. The color scale denotes pore equivalent diameter. The red circle denotes and follows the largest pore identified in the Pre-Testing sample

**Fig. 13:**
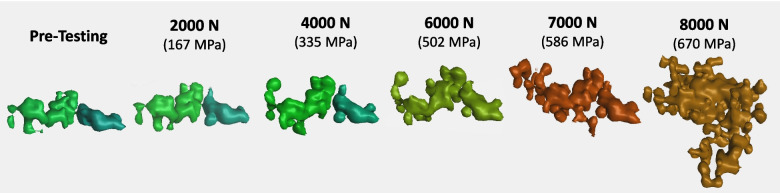
Expanded view of the largest pore highlighted in Fig. 12 as it evolved under loading. The color follows the size scale given in Fig. 12

A 3D magnified view of Layer 2 at each applied load is given in Fig. 12. The detected pores are shaded based on their equivalent size using the color scale in the figure, equivalent to the scale in Fig. 10. The previously described increase in detected pores from the Pre-Testing to the 2000 N frames is clearly seen here. These frames can also be compared to those in Fig. 9, particularly the darker gray spots in the frames of Fig. 9, which show the existence of a population of pores too small to be detected by the algorithm. The largest observed pore that was highlighted in Fig. 8 and Fig. 9 is also located in Layer 2 and is tracked with the red circle. A visual composite image of the largest pore and its evolution under loading is shown in Fig. 13. The Pre-Testing frame shows the 3D geometry of the largest pore along with a sizable neighboring pore. The largest is the green shaded pore on the left possessing an initial equivalent diameter of approximately 249 μm, see Table 4. The neighboring pore is the aqua shaded pore on right and is approximately 209 μm in size. Both pores have an irregular geometry that appears to consist of several interconnected LOF pores in both the build and lateral directions. A cursory survey of the other pores in the Pre-Testing frame in Fig. 12 also indicate that pores larger than the individual ~ 70 μm pores generally have a similar interconnected LOF geometry.

**Table 4:** Evolution of the equivalent diameter for the largest pore with applied load

Applied Load (N)	Pre	2000	4000	6000	7000	8000
Equivalent Diameter (μm)	249	255	256	548	612	658

**Fig. 14:**
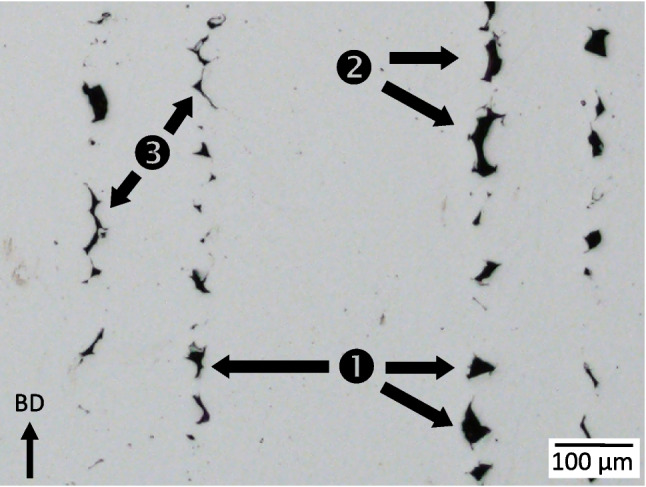
Optical cross-section showing (1) individual LOF pores, (2) pores with a wide interconnection region, and (3) pores with a narrow interconnection region not detectible by the porosity algorithm

As load is applied to the 2000 N and 4000 N levels, these pores appear to evolve in geometry in two ways. First, each increases in equivalent diameter by acquiring neighboring LOF pores with the largest pore increasing approximately 10 μm in equivalent diameter to 256 μm. These new acquisitions appear to be in the build direction, from layers just above or below this defect layer. Second, some interconnection regions are also observed to become more defined with deformation. In both cases the applied load/stress is rather low with behavior governed by elastic deformation. Thus, it is assumed that these observed new interconnections and features already existed and only became detectable by the porosity algorithm due to their increased dimensions under loading. These LOF interconnections are confirmed in the optical image of this parameter set in Fig. 3, highlighted by the blue box, and shown in full scale in Fig. 14. Here, three types of porosity are identified denoted by: () individual LOF pore, () pore with a wide interconnected region between neighboring layers, and () pore with a narrow interconnected region between neighboring layers. While the 4000 N (335 MPa) load is still a few hundred MPa below the continuum yield stress, a number of pores emerge with equivalent diameter approaching the largest pore. It is unclear at this time whether these new, larger pores are; (1) being created via interconnection events from permanent deformation, (2) being newly identified as existing interconnection regions having sufficient dimension to become detectable by the porosity algorithm, or (3) erroneously identified void space interconnection by gray value errors caused by beam hardening effects [60].

**Fig. 15:**
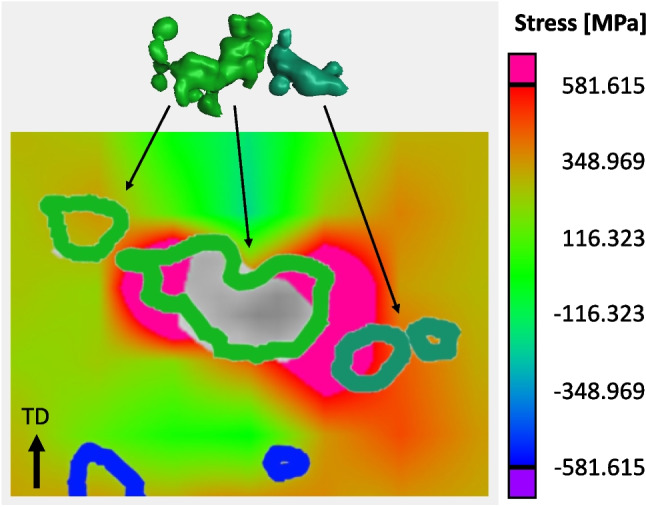
Cross-section of the simulation performed on the 4000 N reconstruction file illustrating the stress concentration locations that would arise in increasing the applied load to 6000 N

Increasing the load to 6000 N (502 MPa) results in a doubling of the largest pore’s equivalent diameter to 548 μm, see Table 4. As observed in the 6000 N frame of Fig. 12, this involves incorporation of the significantly sized neighboring pore through an assumed interconnection event. At this load, the global applied stress is 502 MPa, just under the reported yield stress of 544 MPa. Although the global applied stress is less than the yield stress, local stress concentrations in the vicinity of the pore are likely on the order of or greater than the alloy’s continuum yield stress. Thus, the increase in size or interconnection event is assumed to be plastic or permanent in nature. This hypothesis was tested by employing the 4000 N reconstruction file in finite element simulations to observe the locations and magnitude of stress concentrations relative to the porosity structure. These simulation results are shown in Fig. 15, which shows a cross-section through the largest pore at 4000 N and its closest neighbor. The tensile direction is noted by the arrow. The results show a stress concentration exceeding the continuum yield stress of 531 MPa exists in the space between the two pores indicating their interconnection was probably plastic in nature. This establishes that further evolution of the porosity structure will likely involve interconnection between neighboring pores via permanent deformation mechanisms.

**Fig. 16:**
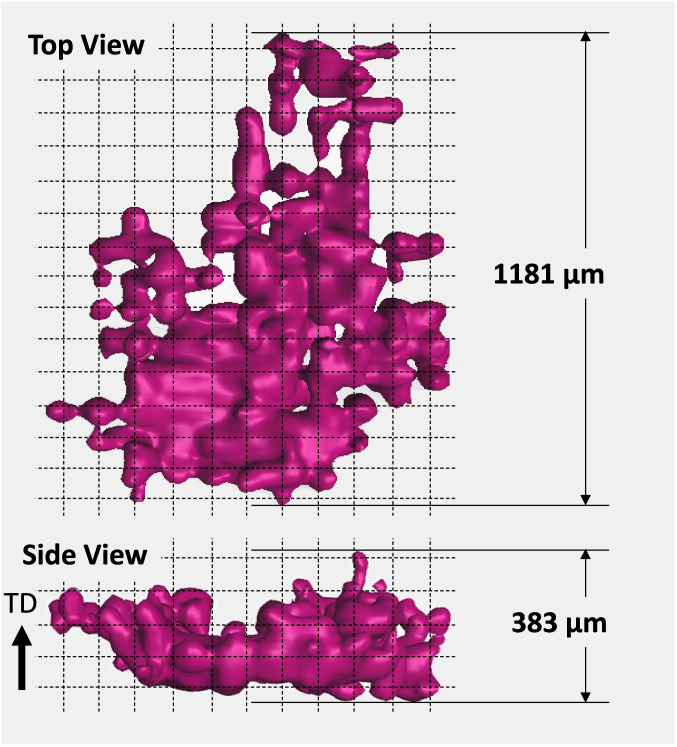
Top and side views of the large pore created at the 8000 N applied load level. The grid size is approximately 100 μm and the tensile direction (TD) is also the build direction (BD)

Increasing the applied load to 7000 N (586 MPa) and 8000 N (670 MPa) resulted in stresses that exceeded the continuum yield stress. At these loads, the largest pore evolved in equivalent diameter, forming an increasingly irregular shape as it interconnected with neighboring pores in both the lateral and tensile directions. It is interesting to note that a significantly sized pore is nucleated at the applied load of 8000 N that did not exist at the previous load increment, see Fig. 16. Here, the pore is viewed from the top and side perspectives where the grid spacing roughly approximates the hatch spacing of 100 μm. Given that the pore geometry appears to be strongly correlated to the array of LOF pores, its formation and growth were likely determined by interconnection events between neighboring pores. Figure 13 and Fig. 16 indicate that pores tend to interconnect more frequently in the lateral direction than in the tensile direction. This suggests the development of plane stress in the intermediate spaces between pores under loading, likely leading to early coalescence and premature fracture initiation from the collective pore volume [1]. This observation aligns with the reduced necking and elongation, up to 50%, observed in the LOF stress–strain behavior.

Failure Analysis.

**Fig. 17:**
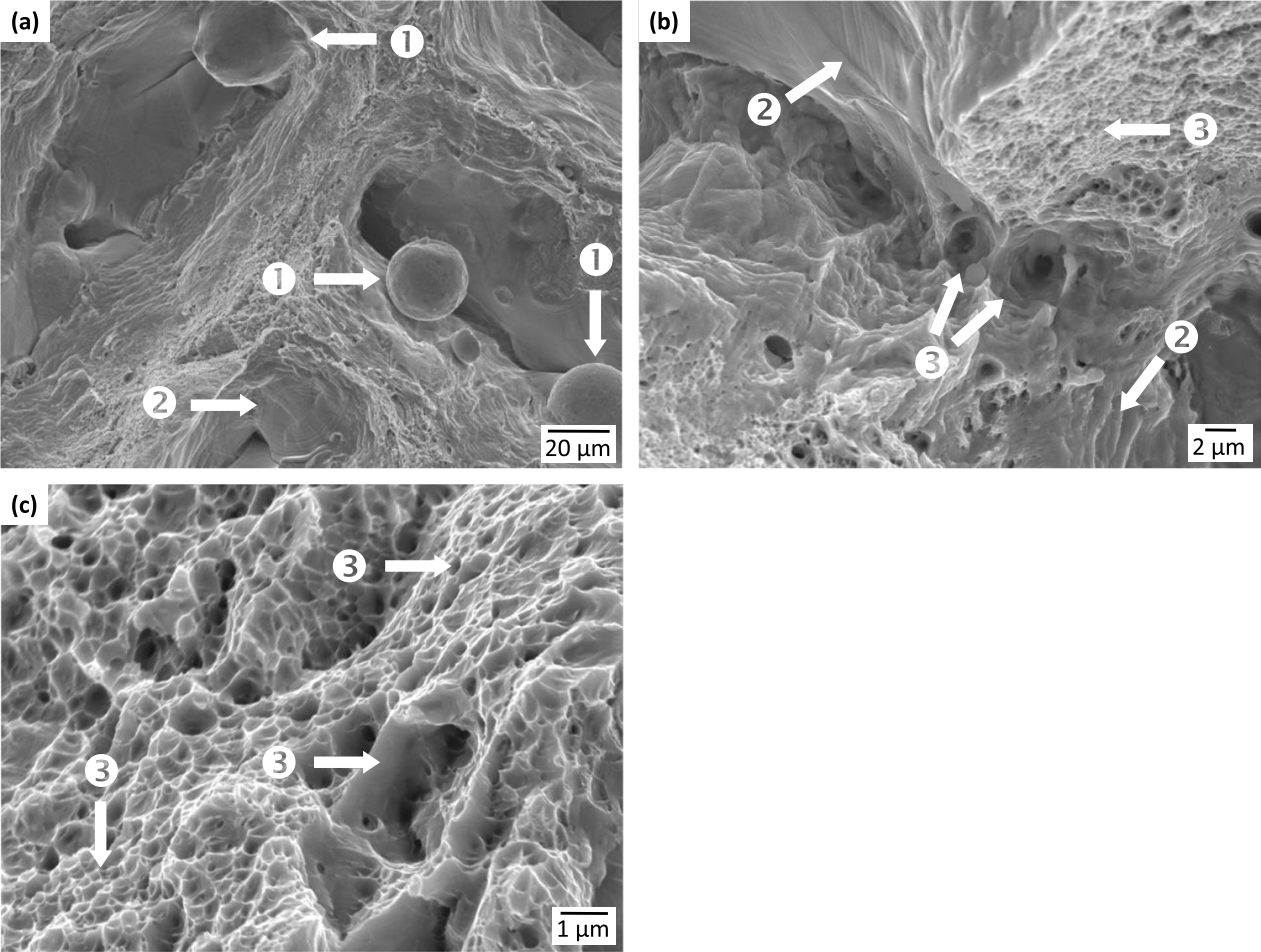
Fracture images of the LOF sample of (a) low magnification near interconnected pores, (b) high magnification near interconnected pores, and (c) high magnification away from pores. The ❶ denotes unfused power particles, ❷ cleavage striations, and ❸ various sized ductile dimples

Fractography based on SEM images and XCT reconstructions was performed to gain insight into the fracture behavior of the LOF sample and its connection to the existing porosity structure. Two SEM images of the fracture surface are given in Fig. 17. Part (a) is a low magnification image taken within the region of a large, interconnected pore. secondary electron mode and shows several LOF pores with associated unfused powder particles, denoted by ❶. Seen in the immediate LOF pore vicinity are striations that represent transitions into cleavage failure, denoted by ❷. At the higher magnification of part (b) the cleavage striations are more defined as well as variable sized dimples near the edges of the interconnected pores, ❸, whose morphology indicate transgranular ductile fracture. Part (c) is from an area away from the pores and shows primarily ductile dimples and transgranular fracture, which is more indicative of the continuum behavior of the sample. Overall, the surface features indicate a mixture of fracture mechanisms with both transgranular ductility and brittle behaviors. This is often referred to as a quasi-cleavage fracture mode [61]. This blended behavior is likely the reason for the reduced elongation observed in the LOF sample.

**Fig. 18:**
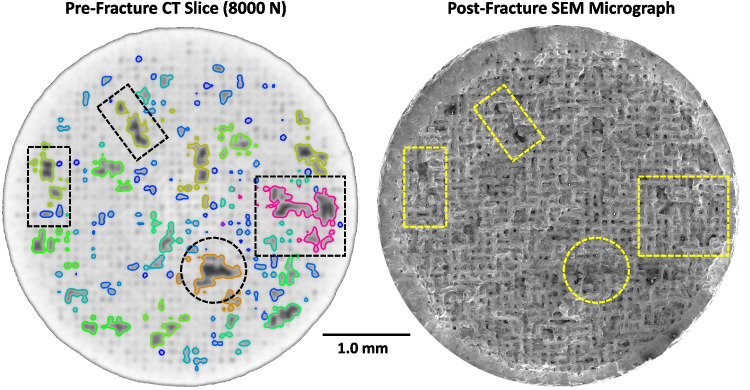
Comparison of the re-fracture XCT slice at 8000 N with the post-fracture SEM image. The dashed shapes are fiducial guide marks between the two images. The labeling of pore size with color in the XCT slice follows the scale in Fig. 12

A more comprehensive understanding of the relationship between the internal porosity and fracture behavior can be made with a direct comparison between the XCT data and the fracture surface, see Fig. 18. On the left-side is a 2D cross-section of the XCT 3D reconstructed region where the specimen failed, Layer #2 identified in Fig. 10. It is taken from the 8000 N load application just before failure. The detected pores are colored by the size scale given in Fig. 12. The array of darker gray spots reveals those pores that there is still a population of pores too small to be detected by the algorithm. They exhibit a complex, interconnected 3D structure in all spatial directions, explaining the apparent discontinuities in this discrete slice. The right-side shows the complementary SEM fracture surface. Four shapes serve as fiducial marks to link identical structural features between the images, clearly linking the pre-failure porosity structure with the resulting fracture surface features. By referring to the failure mechanisms identified in Fig. 17 and the discrete deformation events observed in the LOF stress–strain behavior in Fig. 6, a clearer picture of the failure process emerges. The discrete events seen in the LOF stress–strain behavior most likely result from cleavage failure as neighboring pores undergo interconnection events. This is evidenced by the frequent observation of striations within the immediate vicinity of the large, interconnected pores. Moreover, the local stress concentrations that triggered these events likely arose more rapidly around the porosity structure than in the continuum microstructure. In other regions the fracture surface was mostly represented by ductile dimple features, most likely representing the normal transgranular failure typical of this alloy.

Finally, the six discrete defect layers identified in the 3D porosity analyses presented in Fig. 10 were found to originate from powder particles entrapped in ball screw drive mechanism that controls the build plate’s positional height. This mechanism provides the precise control and repeatability necessary for layer-by-layer construction of a laser powder bed fusion unit. It uses a felt seal to trap and contain powder in the build chamber. Cleaning the mechanism and replacing the felt seal after two build jobs eliminated the issue.

The technique of using AM tailored porosity structures in *in-situ* mechanical/XCT characterization enables a new opportunity to investigate the dynamic processes that occur between microscale voids under applied stress. By capturing these processes as they happen, researchers can better understand material behavior under different loading conditions. The results produced will undoubtably offer new insight into the fundamental understanding of the initiation, growth, and eventual coalescence of microvoids in alloys.

## Conclusions

This work investigated the *in-situ* mechanical/XCT characterization of additively manufactured PBF-LB 316L Stainless Steel with an intended array of micro-scale lack of fusion (LOF) defects. The aim was to develop a technique to investigate the dynamic processes that occur between microscale voids under applied stress and infer how stress concentrations develop between them. The LOF pores were intentionally introduced by varying process parameters. X-ray computed tomography (XCT) was employed to characterize the initial internal defect structure. It was then combined with tensile testing to enable *in-situ* characterization of how the defects evolved under stress. The overall LOF specimen failure manifested as a macroscopically ductile fracture process, with minimal elongation and necking, which was uncharacteristic of relatively defect free conventional, and AM processed 316L stainless steel. Analysis of both pore structure and mechanical behavior was performed and correlated through a direct comparison of stress–strain behavior, reconstructed 3D digital 3D models, scanning electron identification of deformation mechanisms. The results revealed several interesting findings of the LOF porosity and their evolution under loading:

- Porosity populations can evolve under loading in two ways: (1) pores that were not previously identified by the XCT algorithm become detectable when their dimensions increase above the detection threshold, and (2) when local stresses between neighboring pores exceed the yield stress, inelastic deformation mechanisms will permanently interconnect them into a single, larger pore.
- Identified failure mechanisms indicated that pore interconnection events likely occurred by cleavage while the remainder of failure occurred by transgranular fracture.
- Pore interconnection events tended to occur more frequently in the lateral direction than in the tensile direction, suggesting the development of plane stress in the intermediate spaces between pores under loading.
- Pores that did not undergo interconnection events were found to revert to their initial size and population after unloading.
- The porosity structure before failure was correlated to features on the fracture surface with high fidelity.

Combining XCT and tensile testing to enable *in-situ* monitoring of defects and their evolution under load was successful in characterizing the internal porosity and its evolution under load. It also enabled the porosity structure to be correlated to stress–strain behavior and fracture characteristics of sample. The technique is limited to porosity that can be detected by the by the analysis algorithm, which is based on the material scanned and the voxel resolution achievable by the XCT hardware and geometrical set-up. None the less, it is a new tool to enable the insights into the initiation, growth, and eventual coalescence of microvoids. Future investigations can gain more insight with higher resolution through employing higher geometric magnification and/or using alloys with X-ray attenuation factors much lower than 316L SS.

## Data Availability

The data that support the findings of this study are available from the corresponding author upon reasonable request.

## References

[1] Cottrell AH (1953) "Fracture," In: Averbach BL (ed) Proceedings of an international conference on the atomic mechanisms of fracture, Swampscott, Massachusetts MIT Press/Wiley, p. 20.

[2] Rogers HC (1959) The mechanism of crack propagation in ductile metals. Acta Metallurgica 7(11):750–752. 10.1016/0001-6160(59)90184-1

[3] Thomason PF (1990) "The mechanics of microvoid nucleation and growth in ductile metals," Ductile Fract Met Article pp. 30–55. [Online]. Available: https://www.scopus.com/inward/record.uri?eid=2-s2.0-85104300150&partnerID=40&md5=1efcfba6b102228cc98c91a2acf2c839

[4] Lewandowski JJ (2013) Modern fracture mechanics. Phil Mag 93(28–30):3893–3906

[5] Marteleur M, Leclerc J, Colla M-S, Nguyen V-D, Noels L, Pardoen T (2021) Ductile fracture of high strength steels with morphological anisotropy, Part I: Characterization, testing, and void nucleation law. Eng Fracture Mechanics 244:107569. 10.1016/j.engfracmech.2021.107569

[6] Guo Y, Burnett TL, McDonald SA, Daly M, Sherry AH, Withers PJ (2022) 4D imaging of void nucleation, growth, and coalescence from large and small inclusions in steel under tensile deformation. J Mater Sci Technol 123:168–176

[7] Vaughan MW et al (2024) The mechanistic origins of heterogeneous void growth during ductile failure. Acta Materialia 274:119977. 10.1016/j.actamat.2024.119977

[8] Seede R, Johnson K, Noell PJ (2023) Ductile failure and damage localization in Al6061-T6 characterized by in situ X-ray computed tomography and neural network segmentation. Fatigue Fract Eng Mater Struct 46(3):886–894

[9] Lecarme L et al (2014) Heterogenous void growth revealed by in situ 3-D X-ray microtomography using automatic cavity tracking. Acta Mater 63:130–139

[10] Cheng Z et al (2023) Coupled crystal plasticity and micromechanics damage model based on viscoplastic self-consistent theory and X-ray computed tomography. Int J Plast 160:103511

[11] Maire E, Withers PJ (2014) Quantitative X-ray tomography. Int Mater Rev 59(1):1–43. 10.1179/1743280413Y.0000000023

[12] Stock SR (2008) Recent advances in X-ray microtomography applied to materials. Int Mater Rev 53(3):129–181. 10.1179/174328008X277803

[13] Kim FH, Pintar AL, Moylan SP, Garboczi EJ (2019) "The Influence of X-Ray Computed Tomography Acquisition Parameters on Image Quality and Probability of Detection of Additive Manufacturing Defects," J Manuf Sci Eng 141(11). 10.1115/1.4044515 10.1115/1.4044515 PMC8200929 34131380

[14] Withers PJ et al (2021) X-ray computed tomography. Nat Rev Methods Primers 1(1):18. 10.1038/s43586-021-00015-4

[15] Hosokawa A (2010) "Void growth and coalescence studied by X-ray computed tomography".

[16] Hosokawa A, Wilkinson DS, Kang J, Maire E (2013) Onset of void coalescence in uniaxial tension studied by continuous X-ray tomography. Acta Mater 61(4):1021–1036

[17] Weck A, Wilkinson D, Maire E, Toda H (2008) Visualization by X-ray tomography of void growth and coalescence leading to fracture in model materials. Acta Mater 56(12):2919–2928

[18] Azman MA, Le Bourlot C, King A, Fabrègue D, Maire E (2022) 4D characterisation of void nucleation, void growth and void coalescence using advanced void tracking algorithm on in situ X-ray tomographic data. Mater Today Comm 32:103892. 10.1016/j.mtcomm.2022.103892

[19] Landron C, Maire E, Bouaziz O, Adrien J, Lecarme L, Bareggi A (2011) Validation of void growth models using X-ray microtomography characterization of damage in dual phase steels. Acta Materialia 59(20):7564–7573. 10.1016/j.actamat.2011.08.046

[20] Maire E, Zhou S, Adrien J, Dimichiel M (2011) Damage quantification in aluminium alloys using in situ tensile tests in X-ray tomography. Eng Fracture Mechanics 78(15):2679–2690. 10.1016/j.engfracmech.2011.07.004

[21] Balan T, Lemoine X, Maire E, Habraken A-M (2015) Implementation of a damage evolution law for dual-phase steels in Gurson-type models. Mater Design 88:1213–1222. 10.1016/j.matdes.2015.09.075

[22] Pathak N, Butcher C, Adrien J, Maire E, Worswick M (2020) “Micromechanical modelling of edge failure in 800 MPa advanced high strength steels.” J Mechanics Phys Solids 137:103855. 10.1016/j.jmps.2019.103855

[23] Lecarme L et al (2014) Heterogenous void growth revealed by in situ 3-D X-ray microtomography using automatic cavity tracking. Acta Materialia 63:130–139. 10.1016/j.actamat.2013.10.014

[24] Hirano T, Usami K, Tanaka Y, Masuda C (1995) In situ x-ray CT under tensile loading using synchrotron radiation. J Mater Res 10:381–386

[25] Seo D, Toda H, Kobayashi M, Uesugi K, Takeuchi A, Suzuki Y (2015) In situ observation of void nucleation and growth in a steel using X-ray tomography. isij int 55(7):1474–1482

[26] Maire E, Le Bourlot C, Adrien J, Mortensen A, Mokso R (2016) 20 Hz X-ray tomography during an in situ tensile test. Int J Fract 200:3–12

[27] Glinz J, Maurer J, Eckl M, Kastner J, Senck S (2022) "In-situ characterization of additively manufactured continuous fiber reinforced tensile test specimens by X-ray computed tomography," In AIAA SCITECH 2022 Forum p. 1426

[28] Wang L, Zhang W, Li H, Hou C, Ren F (2020) 3D in-situ characterizations of damage evolution in C/SiC composite under monotonic tensile loading by using X-ray computed tomography. Appl Compos Mater 27:119–130

[29] Niu G et al (2022) Internal damage evolution investigation of C/SiC composites using in-situ tensile X-ray computed tomography testing and digital volume correlation at 1000° C. Compos A Appl Sci Manuf 163:107247

[30] Mazars V et al (2017) Damage investigation and modeling of 3D woven ceramic matrix composites from X-ray tomography in-situ tensile tests. Acta Mater 140:130–139

[31] Maurer J, Jerabek M, Salaberger D, Thor M, Kastner J, Major Z (2022) Stress relaxation behaviour of glass fibre reinforced thermoplastic composites and its application to the design of interrupted in situ tensile tests for investigations by X-ray computed tomography. Polym Testing 109:107551

[32] Löffl C, Saage H, Göken M (2019) In situ X-ray tomography investigation of the crack formation in an intermetallic beta-stabilized TiAl-alloy during a stepwise tensile loading. Int J Fatigue 124:138–148

[33] Kafka OL et al (2022) X-ray computed tomography analysis of pore deformation in IN718 made with directed energy deposition via in-situ tensile testing. Int J Solids Struct 256:111943

[34] Yang Q et al (2024) In-situ X-ray computed tomography tensile tests and analysis of damage mechanism and mechanical properties in laser powder bed fused Invar 36 alloy. J Mater Sci Technol 175:29–46

[35] Croom BP, Jin H, Noell PJ, Boyce BL, Li X (2019) Collaborative ductile rupture mechanisms of high-purity copper identified by in situ X-ray computed tomography. Acta Mater 181:377–384

[36] Rueckel J, Stockmar M, Pfeiffer F, Herzen J (2014) Spatial resolution characterization of a X-ray microCT system. Appl Radiation Isotopes 94:230–234. 10.1016/j.apradiso.2014.08.014 10.1016/j.apradiso.2014.08.014 25233529

[37] McCullough EC et al (1976) Performance Evaluation and Quality Assurance of Computed Tomography Scanners, with Illustrations from the EMI, ACTA, and Delta Scanners. Radiology 120(1):173–188. 10.1148/120.1.173 935444 10.1148/120.1.173

[38] Talebinezhad H, Fischer R, Shmatok A, Prorok BC (2023) A straightforward and analytical method for precise X-ray CT magnification correction. Precision Engineering 82:338–349. 10.1016/j.precisioneng.2023.04.004

[39] Kotzem D et al (2021) Impact of single structural voids on fatigue properties of AISI 316L manufactured by laser powder bed fusion. Int J Fatigue 148:106207. 10.1016/j.ijfatigue.2021.106207

[40] Wilson-Heid A, Novak T, Beese AM (2019) Characterization of the effects of internal pores on tensile properties of additively manufactured austenitic stainless steel 316L. Exp Mech 59:793–804

[41] Kim F, Moylan SP, Phan TQ, Garboczi E (2020) Investigation of the effect of artificial internal defects on the tensile behavior of laser powder bed fusion 17–4 stainless steel samples: simultaneous tensile testing and X-ray computed tomography. Exp Mech 60:987–1004 10.1007/s11340-020-00604-6 PMC9813929 36619901

[42] Kim FH, Kim FH, Moylan SP (2018) Literature review of metal additive manufacturing defects. US Department of Commerce, National Institute of Standards and Technology …,

[43] Spierings AB, Schneider MU, Eggenberger R (2011) Comparison of density measurement techniques for additive manufactured metallic parts. Rapid Prototyping J 17(5):380–386

[44] Hansen K, Kristiansen M, Olsen F (2014) Beam shaping to control of weldpool size in width and depth. Phys Procedia 56:467–476

[45] Sibillano T, Ancona A, Berardi V, Schingaro E, Basile G, Lugarà P (2007) Optical detection of conduction/keyhole mode transition in laser welding. J Mater Process Technol 191(1–3):364–367

[46] Tenbrock C et al (2020) Influence of keyhole and conduction mode melting for top-hat shaped beam profiles in laser powder bed fusion. J Mater Processing Technol 278:116514. 10.1016/j.jmatprotec.2019.116514

[47] Rai R, Elmer J, Palmer T, DebRoy T (2007) Heat transfer and fluid flow during keyhole mode laser welding of tantalum, Ti–6Al–4V, 304L stainless steel and vanadium. J Phys D Appl Phys 40(18):5753

[48] David SA, DebRoy T (1992) Current Issues and Problems in Welding Science. Science 257(5069):497–502. 10.1126/science.257.5069.497 17778680 10.1126/science.257.5069.497

[49] King WE et al (2014) Observation of keyhole-mode laser melting in laser powder-bed fusion additive manufacturing. J Mater Process Technol 214(12):2915–2925

[50] Patel S, Vlasea M (2020) Melting modes in laser powder bed fusion. Materialia 9:100591. 10.1016/j.mtla.2020.100591

[51] Trapp J, Rubenchik AM, Guss G, Matthews MJ (2017) In situ absorptivity measurements of metallic powders during laser powder-bed fusion additive manufacturing. Appl Mater Today 9:341–349

[52] Basak A, Das S (2016) Epitaxy and microstructure evolution in metal additive manufacturing. Annu Rev Mater Res 46:125–149

[53] Kim K-T (2022) Mechanical performance of additively manufactured austenitic 316L stainless steel. Nuclear Eng Technol 54(1):244–254. 10.1016/j.net.2021.07.041

[54] Lavery N et al (2017) Effects of hot isostatic pressing on the elastic modulus and tensile properties of 316L parts made by powder bed laser fusion. Mater Sci Eng, A 693:186–213

[55] Shamsujjoha M, Agnew SR, Fitz-Gerald JM, Moore WR, Newman TA (2018) High strength and ductility of additively manufactured 316L stainless steel explained. Metall and Mater Trans A 49:3011–3027

[56] Kurzynowski T, Gruber K, Stopyra W, Kuźnicka B, Chlebus E (2018) Correlation between process parameters, microstructure and properties of 316 L stainless steel processed by selective laser melting. Mater Sci Eng, A 718:64–73

[57] Casati R, Lemke J, Vedani M (2016) Microstructure and fracture behavior of 316L austenitic stainless steel produced by selective laser melting. J Mater Sci Technol 32(8):738–744

[58] Carlton HD, Haboub A, Gallegos GF, Parkinson DY, MacDowell AA (2016) Damage evolution and failure mechanisms in additively manufactured stainless steel. Mater Sci Eng, A 651:406–414

[59] Tolosa I, Garciandía F, Zubiri F, Zapirain F, Esnaola A (2010) Study of mechanical properties of AISI 316 stainless steel processed by “selective laser melting”, following different manufacturing strategies. Int J Adv Manuf Technol 51:639–647

[60] Lifton J, Liu T (2021) An adaptive thresholding algorithm for porosity measurement of additively manufactured metal test samples via X-ray computed tomography. Addit Manuf 39:101899

[61] Kim H, Liu Z, Cong W, Zhang H-C (2017) Tensile fracture behavior and failure mechanism of additively-manufactured AISI 4140 low alloy steel by laser engineered net shaping. Materials 10(11):1283 29120374 10.3390/ma10111283 PMC5706230

